# Effects of berberine hydrochloride on methamphetamine-induced anxiety behaviors and relapse in rats 

**DOI:** 10.22038/ijbms.2020.47285.10884

**Published:** 2020-11

**Authors:** Leila Rezaeian, Hamid Kalalian-Moghaddam, Fahimeh Mohseni, Mehdi Khaksari, Raheleh Rafaiee

**Affiliations:** 1 Department of Addiction Studies, School of Medicine, Shahroud University of Medical Sciences, Shahroud, Iran; 2 Addiction Research Center, Shahroud University of Medical Sciences, Shahroud, Iran; 3 Student Research Committee, School of Medicine, Shahroud University of Medical Sciences, Shahroud, Iran; 4 Department of Physiology, School of Medicine, Shahroud University of Medical Sciences, Shahroud, Iran; 5 Department of Neuroscience, School of Advanced Technologies in Medicine, Mazandaran University of Medical Sciences, Sari, Iran

**Keywords:** Anxiety, Berberine, Methamphetamine, Neuronal cell death, Neuroprotection, Relapse

## Abstract

**Objective(s):**

This research aimed at evaluating the effect of berberine hydrochloride on anxiety-related behaviors induced by methamphetamine (METH) in rats, assessing relapse and neuroprotective effects.

**Materials and Methods:**

27 male Wistar rats were randomly assigned into groups of Control, METH-withdrawal (METH addiction and subsequent withdrawal), and METH addiction with berberine hydrochloride oral treatment (100 mg/kg/per day) during the three weeks of withdrawal. Two groups received inhaled METH self-administration for two weeks (up to 10 mg/kg). The elevated plus maze (EPM) test and open field test (OFT) were carried out one day after the last berberine treatment and relapse was assessed by conditional place preference (CPP) test. TUNEL assay and immunofluorescence staining for NF-κB, TLR4, Sirt1, and α-actin expression in the hippocampus were tested.

**Results:**

After 3 weeks withdrawal, berberine hydrochloride decreased locomotor activity and reduced anxiety-related behaviors in comparison with the METH-withdrawal group (*P*<0.001). The obtained results from CPP showed that berberine significantly reduced relapse (*P*<0.01). Significantly decrease in activation of TLR4, Sirt1, and α-actin in METH-withdrawal group was found and the percentage of TLR4, Sirt1, and α-actin improved in berberine-treated group (*P*<0.001). A significant activity rise of NF-κB of cells in the METH-withdrawal group was detected compared to berberine-treated and control groups (*P*<0.001).

**Conclusion:**

Treatment with berberine hydrochloride via modulating neuroinflammation may be considered as a potential new medication for the treatment of METH addiction and relapse. The histological assays supported the neuroprotective effects of berberine in the hippocampus.

## Introduction

Methamphetamines (METH) are well-known stimulant drugs with a broad range of impact on the central nervous system (CNS) (1). A line of evidence demonstrated that consumption of METH produces long-lasting neurotoxicity to dopaminergic and serotonergic nerve terminals of the hippocampus. The process of METH-caused toxicity has not been identified, but, METH-related neuropathology can be due to oxidative stress, excitotoxicity, hyperthermia, and neuroinflammatory reactions (2), which leads to neuronal degeneration and death (3). The dorsal hippocampus is effective in some types of learning and memory, especially spatial learning; however, ventral hippocampus is possibly effective in brain processes involved in anxiety-induced behaviors (4). 

Several studies have shown that the biomarkers of inflammation, like inflammatory cytokines and acute-phase proteins are increased in hippocampal regions in cases suffering from mood and anxiety disorders (5). METH has a direct effect on endothelial cell features or indirect effect through astrocytes by releasing tumor necrosis factor alpha (TNF-α) and further the pathway of nuclear factor-κB (NF-κB) activation detected in blood-brain barrier impairment (6). Anxiety could activate NF-κB signaling and reduce neural stem-like cells proliferation in the adult hippocampus (7). In previous studies, the association of toll-like receptor-4 (TLR4) with learning and memory processes, stress-related adaptations, and neurodegenerative diseases was observed (8-12). METH-related neuroinflammation has shown to be partly adjusted via TLR4 signaling in the ventral tegmental area (VTA) with the downstream impact of increasing dopamine in the nucleus accumbens (NAc) shell (13).

Chronic METH use also induces the silent mating type information regulation proteins (sirtuins) (13). Sirtuin 1 (Sirt1) as a NAD-dependent protein deacetylase is effective in several biological activities like oxidative stress, metabolism, cell proliferation, and genomic stability (14-17). It protects cells from oxidative stress in several disorders associated with neurodegeneration and metabolic diseases (18).

The actin cytoskeleton-regulatory proteins have shown to be linked with neuronal transmission and morphogenesis and in synaptic plasticity in brain regions mediating fear memory (19). The actin cytoskeleton has been raised as a relapse prevention therapeutic target in METH use (20).

Berberine hydrochloride is an isoquinoline plant alkaloid, with anti-inflammatory (21), anticancer (22), antibiotic (23), anxiolytic (24), antiamnesic (25), analgesic (26), and antidepressant activities (27, 28). The berberine anti-anxiety effect is probably because of the elevated turnover frequency of monoamines in the brain stem as well as a reduced serotonergic system function (24). Berberine can inhibit glutamate receptors and decrease glutamate, 5-hydroxytryptamine, and norepinephrine (NE) amounts (29). Berberine significantly reduced pro-inflammatory cytokine production and apoptosis (21, 30, 31). 

No Food and Drug Administration (FDA)-approved drug is available to treat METH addiction and to prevent relapse (32). This research aimed at finding the impact of administration of berberine hydrochloride on METH-induced anxiety behaviors, relapse, apoptosis, and inflammatory agents such as NF-κB, TLR4, Sirt1, and α-actin expression in the hippocampus. 

## Materials and Methods

***Animals***

Male Wistar rats (n=27, W=200-250 g) were provided by Pasteur Institute (Tehran, Iran) and were placed in light/dark cycles of 12 hr at 21±3 °C, humidity of 60 ± 5%, and food and water were accessible. The experiments were conducted between 8:00 and 15:00 and based on the Health’s Guide of National Institutes for using and care of Laboratory Animals. 

***Experimental protocol***

Twenty seven rats were divided into three groups randomly: Control group of untreated intact animals (n=8), METH-withdrawal group; the animals in this group (n=7) received 14 days of inhaled METH followed by 21 days of drug abstinence, Berberine-treated group; the animals in this group (n=12) received 14 days of inhaled METH and daily oral gavage of berberine (100 mg/kg) (33) during the three-week period of withdrawal.

Two groups received METH by Inhalation Self-Administration Apparatus for two weeks. Methamphetamine hydrochloride (Sigma-Aldrich; Merck Millipore, M8750, USA) was dissolved in distilled water (DW) with the concentration of 1 mg/cc (5 mg/kg) during the first week ,and 2 mg/ml (10 mg/kg) at the second week of addicting period; the process was described in our previous paper (34). Berberine hydrochloride (Sigma-Aldrich; Merck Millipore, Germany) were administered by oral gavage (100 mg/kg) in 21 days of METH withdrawal.

**Figure 1:**
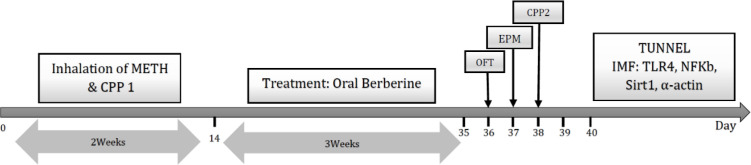
Experimental timeline

METH: methamphetamine; OFT: open field test; EPM: elevated plus maze; IMF: immunofluorescence

At the end of the experiment, anxiety-induced behaviors were measured through the open field test (OFT) and elevated-plus maze (EPM) test one day after last berberine treatment, and relapse was assessed by conditional place preference (CPP) test in all animals of each group. Temperature, humidity, and time remained constant in all experiments. The cell viability and cell apoptosis were assessed in the hippocampus by TUNEL assay, and ​​immunofluorescence staining of hippocampal sections was examined for TLR4, NF-κB, Sirt1 and α-actin expression (Figure 1). Three animals per group for TUNNEL and four animals per group for immunoflurecent staining were used. 

***Inhalation self-administration apparatus***

After training period of self-administration, all animals of METH-withdrawal and berberine-treated rats were put in the self-administration inhaling apparatus (made with Noavaran Sanaye Amouzeshi, Mashhad, Iran) to initiate METH self-administration (15 min/day). The apparatus has different parts as follows; the rats’ cage is made of Plexi Glass, there are 2 levers in the cage, one lever is inactive and the other is active. Pressing on the inactive lever has no consequence, but pressing the active lever by animal will receive the material considered by the researcher, such as food, inhalation drug, or injectable drug and simultaneous red light-emitting diode (LED) is turned on. As the cage is an enclosed space, the METH-induced vapor remains in it for a short time to be inhaled by the animals and will not be scattered in the lab. 

The inhalational self-administration part includes a syringe of 3 ml capacity in which a METH solution is put in the automated injection device. As the animal presses the active lever, the drug is injected into a micro-injection plastic tube at the amount modified by the researcher (METH injection volume is adjustable from 50 to 500 microliters and time intervals between injections are adjustable for 1-10 sec). To have an inhalational model of addiction, METH solution is poured on heated plate to be evaporated. It is placed next to the animal cage. A fan blows the evaporated METH solution into the cage. To prevent rat hot air suffering in the hotplate chamber, a sucker fan is placed in the cage to be simultaneously activated. After METH solution is dripped, the fan starts blowing for 15 sec and then the sucker fan starts to work once more.

Automatic feeding section is a part that after being activated by the researcher, the animal receives a certain amount of food by pressing the active lever. This part of apparatus is used for training the animals to self-administer a drug, as well as to develop addiction. The regulating section of the apparatus is put at the top of it, by which the user can modify the dosage of inhalable drug, which is adjustable in a range from 50 to 500 μl and the intervals of 1 to 10 sec between the injections. Finally, after the end of the experiment, computerized processing unit provides a report on its screen and analyses how many times the inactive and active levers have been pressed for drug intake (34).

***Open field test***

The OFT was used for assessing the anxiety-like behaviors in rats (35). One day after last berberine treatment, the animals were located in the apparatus. It is a Plexi glass box (100 × 100 × 40 cm) with the black bottom separated in sixteen squares as same as in the software for data registration. At first, an animal was placed at the box center followed by recording its motor activities, such as the rate of square crossings in the central square and open area of the box, the duration of staying in central and peripheral regions, and the five min traveled distance using a camera placed above the box.

***Elevated-plus maze test***

It has two open (50 × 10 cm) and two enclosed (50 × 10 × 50 cm) arms and also a central platform (10 ×10 cm) 50 cm over the floor. The rats were first separately located in the center with their heads facing the open arm. They had time for freely exploring the maze through 5 min. The spent time in (sec) and entries to the open arms were measured (36). 

***Conditional place preference task***

The CPP test applied includes three parts, of which 2 and 3 have the same dimensions (30 × 30 × 40 cm). They are differed by a black floor of part two, in which the walls are striped in black and white as well as a lamp in yellow, whereas the third part has black walls and floor and a lamp in red. The first part is considered smaller than the other two (30 × 15 × 40 cm). The entering part is placed in part one. Three parts are separated by guillotine doors. The test is conducted using a five-day model: three individual periods, such as familiarization, conditioning, and test periods.

Familiarization: At the 1^st^ day (prior to making addicting model), the rat was located in apparatus for 15 min with an open entrance and it was able to move freely. Simultaneously, we noted the time spent in compartments two and three. The time spent in each room was measured, and the preferred room is the room in which rats stayed for more time and the other one was the non-preferred room. 

Conditioning: Between the second and fourth day, the rats were treated with inhaled METH, and confined to the non-preferred room for 45 min. After 6 hr, following the DW use in the apparatus, the rats were located in the preferred chamber again within 45 min, while the gates were closed. For the 2^nd^ day, a different procedure from the used one during the first day was applied. At first, animals received DW, and after 6 hr, METH was inhaled. The 3^rd^ conditioning day was the same as the 1^st^ one.

Test: At the day five, CPP task was conducted for the first time with open door. A rat was located in compartment one, in which it was capable of moving in 15 min in all parts. At the same time, the period duration spent in the 2^nd^ and 3^rd^ compartments was recorded. Any change in preference was assessed through comparing the spent time during the examination day in the compartment for administrating METH and the box throughout the preconditioning stage; data was introduced as mean±SD. Familiarization, conditioning and the first CPP test (CPP 1) were performed in first 5 day of METH addiction. Then, after berberine administration, the CPP 2 test was repeated to evaluate relapse (Figure 1).

***Immunofluorescence***

A day after the last behavioral test, an intraperitonealy injection of mixture of ketamine 100 mg/kg and xylazine 10 mg/kg was used for rats deep anesthetizing (37). After making an incision and lifting the anterior chest wall away, the needle was inserted in the left ventricle. The transcardial perfusion of animals with intracardiac injection of 0.9% saline (200 cc) and 4% paraformaldehyde (PFA) in 0.1 M phosphate buffer solution (PBS) was performed and then brains was removed and postfixed in 4% PFA for a night (38). Following conventional paraffin embedding and serial section (5 μm), immunohistochemistry staining was carried out after incubating in primary antibody (Anti-TLR4 antibody: ab22048, Abcam, Germany; Anti-Sarcomeric Alpha Actinin antibody: ab137346, Abcam, Germany; Anti-SIRT1 antibody: ab189494, Abcam, Germany; Anti-NF-κB p65 antibody, Abcam, Germany) for a night at 4 °C and Fluorescein (FITC)-conjugated secondary antibody (Goat Anti-Mouse IgG H&L (FITC), ab6785, Abcam, Germany) for 2 hr. An Olympus FluoView FV1200 microscope (Japan) was used. The quantitative assessment of staining was performed by analySIS software (analySIS 3.0; Soft Imaging System), and the mean value from 25 fields was applied for obtaining a single score for each animal. 

***TUNEL staining***

A day after the last behavioral test, rats were anesthetized following the same protocol as immunofluorescence was performed and brains were removed. *In Situ* Cell Death Detection Kit (Roche, Mannheim, Germany) was used for TUNEL staining based on the instructions provided by producer. In summary, the paraffin-embedded hippocampus of rats (n=3 per group) were incubated in xylol for deparaffinization, dehydrated in a series of alcohols, rinsed in PBS, and permeabilization was performed with 10 mM proteinase K in 30 min at room temperature. Subsequently, washing and incubating the sections was performed using 3% H_2_O_2_ in methanol for 10 min in a dark environment for blocking the activity of endogenous peroxidase, which may possibly lead to false findings. Following adding the mixture of TUNEL reaction, the section incubation was performed in 1 hr at 37 °C in an appropriate humid box followed by washing with PBS and visualizing by converter-POD for 30 min at 37 °C in a dark environment under moderate humidity. They were rinsed by PBS and incubated with 50–100 μl of 0.05 % 3, 3′-diaminobenzidine (DAB) as a chromogen within 10 min. Following PBS washing, they were mounted using a cover slip. Through a light microscope, the TUNEL-positive cells rates were calculated on a transect (400 μm long, 0.160 mm^2^) in hippocampus. All procedures for counting were executed blindly.

***Data analysis***

All data analysis was performed using the GraphPad Prism software (Prism for Windows, version 5.0, GraphPad Software Inc., San Diego, CA, USA). The Kolmogorov–Smirnov test confirmed the normal distribution of each dataset. The behavioral and histological findings were studied through variance analysis (ANOVA) and Tukey’s *post hoc* tests. The values presented as mean±SD and *P*-value of smaller than 0.05 was regarded significant. 

## Results

***Effects of berberine on anxiety in the OFT***

Motor activity was defined as the whole distance traveled in OFT, which significantly elevated in METH-withdrawal rats than the control group. Treatment with berberine significantly decreased METH-induced hyperactivity than the METH-withdrawal group in the total distance (*P*<0.01). In comparison with berberine-treated (1352±403.2 cm) and control groups (1350 ±400.1 cm), total distance moved in OFT significantly increased in METH-withdrawal group (1753±284.1 cm). 

**Figure 2:**
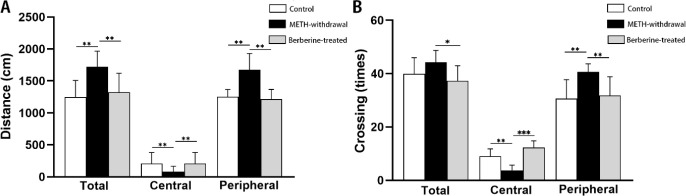
Effect of berberine treatment on locomotion activity following METH addiction in open field test. (A) Total distance moved and distance moved in central and peripheral zone (cm), (B) Total number of crossings and number of crossings in central and peripheral zone. (Control group, n=8; METH-withdrawal group, n=7; Berberine-treated group, n=12). Data are mean±SD; (*P<0.05, **P<0.01, ***P<0.001)

As illustrated in Figure 2A, one-way ANOVA showed that distance moved in the central zone in the rats receiving METH (80.43 ± 85.69 cm) significantly reduced compared to the control (207±172.3 cm) and berberine-treated (205±170.4 cm) groups (F _(2,20) _= 6.554, *P*<0.01). Moreover, distance moved in the peripheral zone in the rats receiving METH (1673±257.4 cm) significantly enhanced compared to the control (1164±466.5 cm) and berberine-treated (1166±468.1 cm) groups (*P*<0.01). The rats of METH-withdrawal group showed significantly increased crossings (total crossings, 54.71±13.15, *P*<0.05; peripheral crossings, 49.00±9.23, *P*<0.01) than the berberine-treated (total crossings, 43.88±15.76; peripheral crossings, 34.88±25.63) group. The peripheral and total crossings in the control group were 44.25±17.79 and 29.25±27.55, respectively. In comparison with berberine-treated (12.63±6.11) and control groups (9.75±6.67), METH-withdrawal group (5.71±6.15) has less crossings in the central zone in OFT (F _(2, 20)_ =3.088, *P*<0.01) (Figure 2A).

***Impact of berberine on anxiety in the EPM***

**Figure 3:**
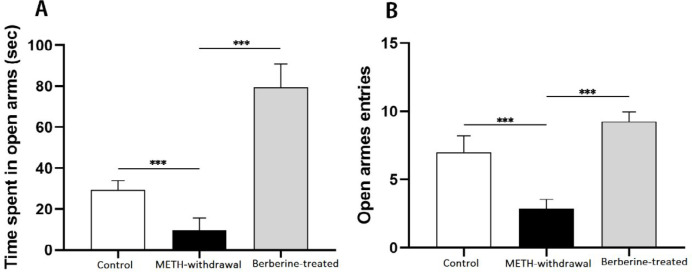
Effect of berberine treatment on behavioral measures following METH addiction in the elevated plus maze. (A) Time spent in open arms (sec), (B) Number of open arms entries. (Control group, n=8; METH-withdrawal group, n=7; Berberine-treated group, n=12). Data are mean±SD; (***P<0.001). METH: methamphetamine

The most common factors for assessing anxiety reflect avoidance of open arms, like the total time left on open arms and entries rate to open arms. One-way analysis of variance revealed that the time spent in the open arms was lower following METH administration (9.71±5.99 sec) than the control group (29.25±4.62 sec, F _(2, 18)_=147.8, *P*<0.001). As shown in Figure 3A, the spent time was significantly enhanced on the open arms after berberine administration (79.50±11.33 sec, *P*<0.001) than the METH-withdrawal group. The entries frequency in the open arms was significantly reduced after the administration of METH (2.85±0.69) compared to the control group (7±1.19). For open arm frequency, berberine-treated (9.25±0.70) group showed significantly more times compared to METH-withdrawal group (F _(2, 20)_= 94.99, *P*<0.001, Figure 3B). 

***Effects of berberine on relapse in the CPP***

**Figure 4:**
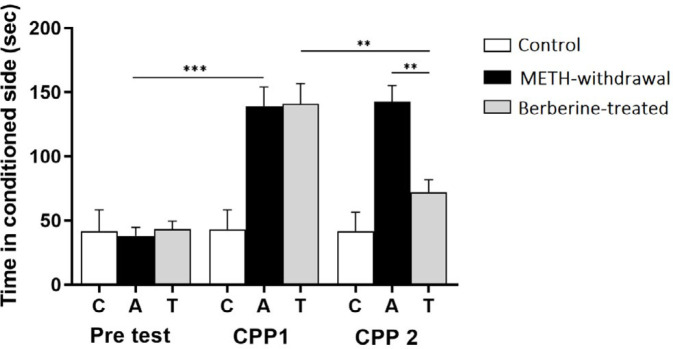
Effect of berberine treatment on conditioned place preferences following METH addiction in the CPP. (C: Control group, n=8; A: METH-withdrawal group, n=7; T: Berberine-treated group, n=12). Data are mean±SD; (**P<0.01, ***P<0.001)

METH: methamphetamine; CCP: conditional place preference

METH-related CPP indicated biased results since the animals preferred the METH-paired place than the vehicle-paired place in a classical conditioning experiment. METH-related dopamine secretion in the nucleus accumbens was regarded as essential and adequate for METH-related CPP. Figure 4 indicates a significantly elevation of the time spent in the METH-paired place in CPP 1 test day (control: 41.71±14.77 sec; METH-withdrawal: 142.5±12.77 sec berberine-treated: 72.00±9.74 sec) than the pretest (control: 43.11±16.78 sec; METH-withdrawal: 38.00±6.69 sec; berberine-treated: 43.17±6.33 sec) in METH-withdrawal rats (*P*<0.001). Moreover, berberine treated rats spent significantly less time in the CPP 2 test day (control: 43.14±14.77 sec; METH-withdrawal: 139.0±15.10 sec; berberine-treated: 72.00±9.74 sec) compared to METH-withdrawal group and CPP 1 test (*P*<0.01).

***Effects of berberine on TLR4, Sirt1, α-actin and NF-*** ***κB*** ***expression in hippocampal tissue in rats***

**Figure 5:**
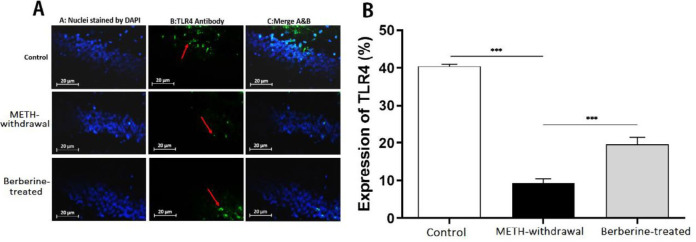
Immunoflurecent staining of TLR4 in the hippocampus of rats. (A) Nuclei staining by DAPI and antibody to TLR4 and merge of them from each group (magnification, ×400). (B), The percentage of positive reaction in each group. Red arrows point to TLR4 positive cells. Scale Bars: 20 µm. Ratio of TLR4-positive neurons is displayed as the mean±SD (n=4 per groups); (***P<0.001)

**Figure 6:**
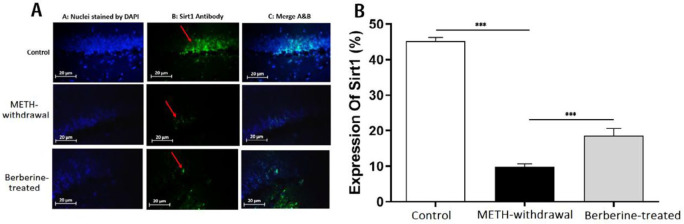
Immunofluorescence staining of Sirt1 in the hippocampus of rats. (A) Nuclei staining by DAPI and antibody to Sirt1 and merge of them from each group (magnification, ×400). (B), The percentage of positive reaction in each group. Red arrows point to Sirt1 positive cells. Scale Bars: 20 µm. Ratio of Sirt1-positive neurons is displayed as the mean±SD (n=4 per groups); (***P<0.001)

**Figure 7:**
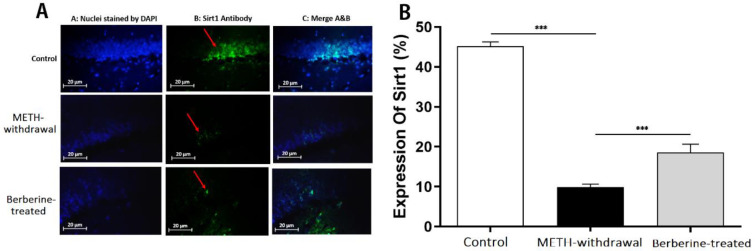
Immunofluorescence staining of α-actin in the hippocampus of rats. (A) Nuclei staining by DAPI and antibody to α-actin and merge of them from each group (magnification, ×400). (B), The percentage of positive reaction in each group. Red arrows point to α-actin positive cells. Scale Bars: 20 µm. Ratio of α-actin positive neurons is displayed as the mean±SD (n=4 per groups); (***P<0.001)

**Figure 8:**
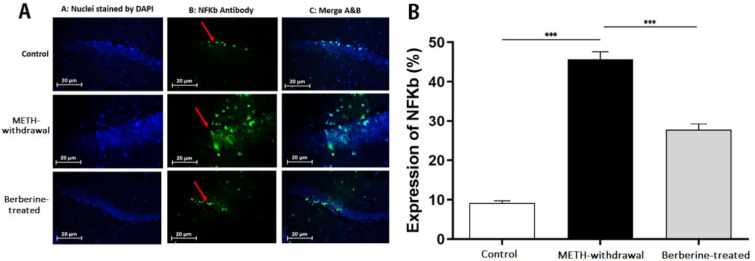
Immunofluorescence staining of NF-κB in the hippocampus of rats. (A) Nuclei staining by DAPI and antibody to NF-κB and merge of them from each group (magnification, ×400). (B), The percentage of positive reaction in each group. Red arrows point to NF-κB positive cells. Scale Bars: 20 µm. Ratio of NF-κB positive neurons is displayed as the mean±SD (n=4 per groups); (***P<0.001). NF-κB, Nuclear factor-κB

Immunofluorescence staining was applied for detecting the TLR4, Sirt1, α-actin, and NF-κB expression in the rat hippocampus at day 1 after the last behavioral tests. Results showed a decrease in the percentage of TLR4 in the METH-withdrawal group (9.28±2.36) compared to the control group (40.48±1.07), while the percentage of TLR4 improved in the berberine-treated group (19.60±3.74) in comparison with METH-withdrawal group (*P*<0.001, Figure 5). The comparison of the percentage of Sirt1 in the METH-withdrawal (9.89±1.47), control (45.14±2.28) and berberine-treated (18.55±4.21) groups showed a significant reduction in the percentage of Sirt1 in METH-withdrawal group and improvement in berberine-treated group (*P*<0.001, Figure 6). The expression of α-actin was significantly increased in METH-withdrawal group (11.23±2.50) in comparison with the control group (46.62±4.21), and berberine significantly improved the expression of α-actin compared to METH-withdrawal group (25.54± 3.04, *P*<0.001, Figure 7). Figure 8 indicates a significant increase of NF-κB activity in METH-withdrawal group (27.77±2.99) compared to control group (9.24±0.95), and berberine decreased NF-κB activity (45.72±3.77, *P*<0.001).

***Effects of berberine on TUNEL staining of the hippocampus in rats***

**Figure 9:**
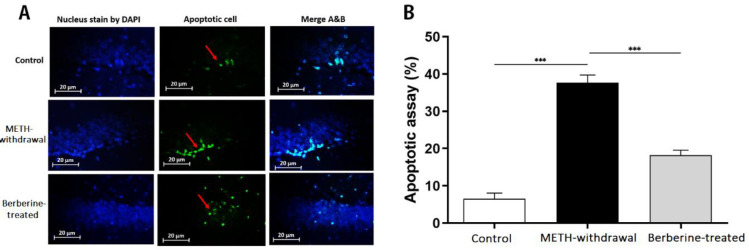
TUNEL staining in the hippocampus of rats. (A) Nuclei staining by DAPI and apoptotic cells and merge of them from each group (magnification, ×400). (B), The percentage of positive reaction in each group. Red arrows point to apoptotic cells. Scale Bars: 20 µm. Ratio of apoptotic cells is displayed as the mean±SD (n=3 per groups); (***P<0.001)

As illustrated in Figure 9, TUNEL staining results showed that TUNEL-positive cells in the hippocampus of rats in METH-withdrawal group (37.65±4.17) was significantly increased in number in comparison with the control group (6.52±3.10, *P*<0.001). Berberine exerted a significant decrease in TUNEL-positive cell rates compared to METH-withdrawal group (18.24±2.67, *P*<0.001). 

METH: methamphetamine

TLR4: toll-like receptor-4

## Discussion

The berberine hydrochloride effectiveness in addition to its neuroprotective effect was evaluated on anxiety-related behaviors and relapse induced by METH in rats. The major findings obtained from the present study are as follows: (1) METH induced an increase in anxiety-related behaviors; (2) Berberine administration to the rats decreased METH-induced anxiety-related behaviors; (3) Berberine administration to the METH-addicted rats decreased relapse; (4) METH led to histological injuries in the hippocampus, while berberine improved METH-induced histological changes in the rats. Of note, our findings are in accordance with the findings which showed that consumption of METH led to anxiety-induced behaviors and tissue damages (39, 40). In addition, the present study provided evidence in favor of the beneficial effect of berberine administration (100 mg/kg) on decreasing the anxiety-induced behaviors of the rats such as decreasing traveled distance, increasing the time spent in central zone in the METH-addicted rat, which was assayed by OFT, and also increased total time spent on open arms and the rate of entries to open arms in EPM. Our findings showed that berberine administration may decrease significantly the amount of time spending in the METH-paired room in CPP task in METH-addicted rats. Furthermore, the present study has explored the histologic change after the administration of berberine to the METH-addicted rats. Interestingly, apoptosis significantly decreased in hippocampus neurons of METH-addicted rats, which was treated with berberine. The obtained findings are consistent with other results in animal models of cerebral pathologies, demonstrating that berberine is able to decrease brain tissue damage as well as neurological impairments (41-44).

The berberine impacts were then measured on the TLR4 and NF-κB participating in the cerebral inflammation. The TLR4 signaling pathway is adjusted with MyD88 activating NF-κB, which leads to the upregulating several pro-inflammatory genes such as cytokines, chemokines, cyclooxygenase 2 (COX-2), and inducible nitric oxide synthase (iNOS). Such pro-inflammatory mediators activate NF-κB, producing a positive feedback loop for amplifying inflammatory signals (45). Berberine attenuated TLR4 and NF-κB expression (46, 47) and also abrogated elevation in inflammatory mediators, such as COX-2, iNOS, interleukin (IL)-6, and macrophage-inflammatory protein 2 (MIP-2) (48-51). The obtained results are consistent with research indicating that berberine decreased tissue impairment via suppressing the TLR4 and NF-κB activations in METH-addicted rats. 

Mounting investigations have shown the effect of berberine, SIRT1, oxidative stress, and inflammation response, respectively (52, 53). Berberine attenuated oxidative stress in mice with diabetes partly by miR-106b/SIRT1 pathway and the islets activity that can be effective to reduce the cardiovascular risk factors (53) and encephalopathy improvement via the SIRT1/ER stress pathway in diabetes (54). Zhu *et al.* (2013) demonstrated that berberine could protect hepatocytes against H_2_O_2_-induced cellular apoptosis by Sirt1 (55). In oxidative stress, Sirt1 exhibited antioxidative function via upregulating the expression of antioxidant enzymes (56). Sirt1 is extensively expressed in the brain and is associated with brain integrity regulating properties maintenance like oxidative stress and neuronal degeneration (57-59). The present findings showed that berberine inhibited apoptosis caused by METH through the Sirt1 activation.

The obtained results do not clarify the mechanism of inhibiting neuronal apoptosis by berberine. Because berberine can suppress extrinsic apoptosis by inhibition of TLR4 signaling as myeloid differentiation factor (MyD)-88 is able to bind Fas-associated death domain protein (FADD) for triggering apoptosis by caspase-8 activation (60). More studies should be performed for clarifying the mechanisms of berberine to modulate inflammation for influencing neuronal apoptosis.

## Conclusion

Berberine administration at 100 mg/kg within 3 weeks could improve anxiety-related behaviors and reduce relapse in METH-addicted rats. The histological assays supported the neuroprotective effects of berberine in the hippocampus. Based on the findings, berberine is a possible therapeutic agent for METH addiction and relapse.
